# Prognostic performance of Hong Kong Liver Cancer with Barcelona Clinic Liver Cancer staging systems in hepatocellular carcinoma

**DOI:** 10.1186/s12876-024-03387-5

**Published:** 2024-09-18

**Authors:** Mohamed Kohla, Reham Ashour, Hossam Taha, Osama El-Abd, Maher Osman, Mai Abozeid, Sally Waheed ELKhadry

**Affiliations:** 1 Department of Hepatology and Gastroenterology, National Liver Institute, Menoufia University, Shebin El-Kom, 32511 Egypt; 2 Department of Diagnostic Medical Imaging and Interventional Radiology, National Liver Institute, Menoufia University, Shebin El-Kom, 32511 Egypt; 3 Department of Hepatopancreatobiliary surgery, National Liver Institute, Menoufia University, Shebin El-Kom, 32511 Egypt; 4 Department of Epidemiology and Preventive Medicine, National Liver Institute, Menoufia University, Shebin El-Kom, 32511 Egypt

**Keywords:** Hepatocellular carcinoma, Staging systems, Hong Kong Liver Cancer, Barcelona Clinic Liver Cancer, Prognostic performance, Egypt

## Abstract

**Background:**

Accurate staging is necessary for predicting hepatocellular carcinoma (HCC) prognosis and guiding patient management. The Barcelona Clinic Liver Cancer (BCLC) staging system has limitations due to heterogeneity observed among patients in BCLC stages B and C. In contrast, the Hong Kong Liver Cancer (HKLC) staging system offers more aggressive treatment strategies.

**Aim:**

To compare the prognostic performance of HKLC and BCLC staging systems in Egyptian patients with HCC.

**Methods:**

We conducted a retrospective study at the National Liver Institute, Menoufia University, Egypt, on 1015 HCC patients. Data was collected from patients’ medical records over 10 years (from 2008 to 2018). The BCLC and HKLC stages were identified, and Kaplan-Meier survival analysis was used to compare patients’ overall survival rates within each staging system. Additionally, we evaluated the comparative prognostic performance of the two staging systems.

**Results:**

Hepatitis C was identified as the underlying etiology in 799 patients (78.7%), hepatitis B in 12 patients (1.2%), and non-viral causes in 204 patients (20.1%). The survival analysis demonstrated significant differences across the various stages within both the BCLC and HKLC systems. The receiver operating characteristic (ROC) curves indicated a marginally superior performance of the HKLC system in predicting survival at 1, 2, and 3 years compared to the BCLC system. Furthermore, the HKLC staging provided a slightly enhanced prognostic capability, particularly for patients classified under BCLC stages B and C, suggesting a potential survival benefit.

**Conclusion:**

HKLC classification had a slightly better prognostic performance than BCLC staging system and may offer a survival advantage for certain patients with HCC in BCLC stage B and C HCC cases.

**Supplementary Information:**

The online version contains supplementary material available at 10.1186/s12876-024-03387-5.

## Introduction

Primary liver cancer is the sixth most commonly diagnosed cancer and the third leading cause of cancer death worldwide in 2020. Hepatocellular carcinoma (HCC) is the dominant type comprising 75-85% of cases [1]. The incidence rates have increased in recent decades with the highest rates observed in Asia and Africa [2]. In Egypt, HCC is one of the most challenging health problems as it represents the fourth common cancer and the leading cause of cancer-related mortality and morbidity [3]. Up to 90% of HCC cases have a cirrhotic liver, but it may also arise without cirrhosis, most commonly in patients with chronic hepatitis B or Non-alcoholic fatty liver disease (NAFLD) [4–7].

Prognostic assessment in HCC patients remains extremely difficult due to the complex interaction of tumor characteristics with the degree of liver dysfunction, patient health status and available treatment options. Several staging systems have been proposed to estimate the prognosis of HCC patients [8].

The Barcelona Clinic Liver Cancer (BCLC) staging system is the most widely applied HCC staging system that has been extensively validated. It is used to guide stage-appropriate treatment and prognostic prediction. Major leading international liver study groups such as the American Association for the Study of Liver Diseases (AASLD) and the European Association for the Study of Liver (EASL) have recommended the BCLC staging system for HCC management [5, 9]– [11]. Despite its popularity, the BCLC staging system has some limitations mainly related to the heterogeneity of BCLC stages B and C patients in respect to tumor burden and liver function [12]. The BCLC approach tends to compromise the application rate of surgical and locoregional therapies in selected patients with BCLC B and C stages especially with recent advances in the surgical and radiological techniques [13].

A group of liver experts developed the Hong Kong Liver Cancer (HKLC) staging system in order to provide more aggressive treatment guidance for Asian HCC patients. According to the HKLC classification, tumor multicentricity or intrahepatic vascular invasion doesn’t contraindicate surgical resection or trans arterial chemoembolization. In addition, advanced liver disease (Child C) and early tumor without extrahepatic vascular invasion or metastases leave patients eligible for liver transplantation [14, 15].

The HKLC staging system can be used both as a prognostic score and as a staging system for treatment assignment. Compared to BCLC classification, HKLC system better stratifies patients assigned to BCLC intermediate and advanced stages resulting in better survival outcomes. The pitfall of HKLC staging system is the lack of solid external validation in non-Asian populations with clinical, biological and etiological heterogeneity since it was developed at a single Asian center that principally treats patients with hepatitis B virus (HBV) infection [8, 16]. So, it’s important to study the prognostic performance of HKLC staging system in different countries where there are more heterogeneous causes of HCC.

## Materials and methods

### Study design

This retrospective cohort included 1015 HCC patients who attended the multidisciplinary HCC clinic over a 10-year period (from 2008 to 2018) at the National Liver Institute, Menoufia University, Egypt.

### Inclusion and exclusion criteria

Patients with a confirmed diagnosis of HCC according to the AASLD Practice Guidelines [17] were included, while those with incomplete records or other primary malignancies were excluded.

### Data collection

Data were collected from patient records, including demographic details, clinical characteristics, laboratory findings, imaging results, Child-Turcotte-Pugh (CTP) class, performance status, tumor characteristics, and treatment modalities for HCC.

### Data analysis

The BCLC and HKLC stages were determined using the collected data. Overall survival (OS) was defined as the period from the initial diagnosis of HCC to the date of death or last follow-up. The BCLC and HKLC staging systems were compared by calculating the median OS for all patients treated under each classification. The outcomes of patients with BCLC stage B and C HCC treated according to BCLC recommendations were compared with those treated according to HKLC guidelines [5, 14].

### Statistical analysis

Statistical analysis was conducted using SPSS version 22 (Armonk, NY: IBM Corp.). Quantitative data were presented as mean, standard deviation (SD), and range, while qualitative data were reported as frequency and percentage. Kaplan-Meier survival analysis was employed to assess survival rates. The Cox regression model was utilized to compute the adjusted hazard ratio and 95% confidence intervals for the effects of various risk factors on survival. The risk ratio (RR) quantified the likelihood of an event occurring in an exposed group relative to a non-exposed comparison group. Confidence intervals (CI) provided estimates of the population parameters, with the proportion of intervals containing the true parameter value reflecting the specified confidence level. Two-sided confidence limits form a confidence interval, while one-sided limits are referred to as lower or upper confidence bounds. The receiver operating characteristic (ROC) curve was used to evaluate the overall effectiveness of tests, with larger areas under the ROC curve indicating better test performance. The Delong test was applied to compare ROC curves and assess the discriminatory ability of different staging systems in predicting survival (18). Statistical significance was defined as a p-value less than 0.05.

## Results

A total of 1015 cirrhotic patients with HCC were included in the study. The baseline descriptive data of these patients were presented in Table 1. Their mean age was 58.65 ± 7.95 and 83.5% of them were males. All patients had liver cirrhosis, and the underlying etiology was mainly hepatitis C virus infection (78.7%) with 71.3% having CTP A and 75.6% with an Eastern Cooperative Oncology Group score of 0. The results of various baseline laboratory parameters were also summarized in Table 1.

**Table 1:** Baseline demographic, clinical criteria, lab, and tumor characteristics of all patients

Baseline demographic, clinical criteria, performance status and Child Pugh class of all patients.				
Studied variable			Frequency	Percent (%)
**Gender**		Male	848	83.5
		Female	167	16.5
**Age**		Mean ± SD Median (Min - Max)	58.65 ± 7.95 58 (20–89)	
**Smoking**		No	489	48.2
		Yes	454	44.7
		EX	72	7.1
**Alcohol consumption**		No	1015	100
**Diabetes mellitus**		No	719	70.8
		Yes	296	29.2
**Hypertension**		No	803	79.1
		Yes	212	20.9
**Ascites**		No	837	82.5
		Yes	178	17.5
**Splenomegaly**		No	251	24.7
		Yes	737	72.6
		Splenectomy	27	2.7
**Underlying aetiology**		Hepatitis C virus (HCV)	799	78.7
		Hepatitis B virus (HBV)	12	1.2
		Non-viral	204	20.1
**Performance Status**		**0**	767	75.6
		**1**	215	21.1
		**2**	22	2.2
		**3**	11	1.1
**Child Pugh Class**		**A**	724	71.3
		**B**	253	25
		**C**	38	3.7
**Baseline laboratory findings of all patients.**				
**Studied variable**			**Mean ± SD**	**Median (Min-Max)**
**Total Bilirubin (mg/dl)**			1.42 ± 1.09	1.2 (0.1–14)
**Direct Bilirubin (mg/dl)**			0.72 ± 0.89	0.5 (0.01-10)
**Alanine Aminotransferase (U/L)**			47.39 ± 32.63	38 (4-250)
**Aspartate Aminotransferase (U/L)**			59.49 ± 41.42	50 (4-300)
**Alkaline Phosphatase (U/L)**			166.20 ± 123.95	134 (10–741)
**Gamma-glutamyl transferase (U/L)**			95.74 ± 114.68	72 (20–765)
**Albumin (g/dl)**			3.51 ± 1.56	3.5 (1.6–5.7)
**Prothrombin concentration (%)**			75.05 ± 15.56	76 (33–101)
**International normalized ratio (INR)**			1.22 ± 0.21	1.2 (0.8–2.9)
**Hemoglobin level (g/dl)**			12.29 ± 1.92	12.40 (7-17.80)
**Total leucocytic count (X 10**^**3**^**/cmm)**			5.64 ± 2.44	5.20 (1.40–18.8)
**Platelet count(X 10**^**3**^**/cmm)**			125.02 ± 68.97	110 (10–622)
**Urea (mg/dl)**			34.35 ± 15.41	31 (8-132)
**Creatinine (mg/dl)**			0.91 ± 0.25	0.9 (0.3–3.1)
**Alpha-fetoprotein (ng/ml)**			2305.24 ± 18570.72	50 (0.2-514180)
**Baseline tumor characteristics of all patients.**				
			**Frequency**	**Percent (%)**
**Lesion Number**:	**Single**		555	54.7
	**Multiple**		420	41.4
	**Diffuse**		40	3.9
**Site of nodule (s)**:	**Unilobar**		782	77
	**Left lobe**		217	21.3
	**Right lobe**		565	55.7
	**Bilobar**		233	23
**Size of nodule (s)**:	**≤ 2**		58	5.7
	**3–5**		439	43.3
	**> 5**		518	51
**Vascular invasion**	**No**		857	84.4
	**Yes**		158	15.6
**Different sites of vascular invasion**	**Main PVT**		63	6.2
	**RT segmental PVT**		39	3.8
	**RT PVT**		22	2.2
	**LT PVT**		15	1.5
	**LT segmental PVT**		14	1.4
	**PVT & SV**		1	0.1
	**PVT & SV & SMV**		1	0.1
	**PVT & SMV & IVC**		1	0.1
	**PVT & HV & IVC**		1	0.1
	**Rt HVT**		1	0.1
**Extrahepatic Metastasis**	**N**		972	95.7
	**Y**		43	4.3
**Site of extra hepatic metastasis**	**Lymph node**		18	1.8
	**Bone metastasis**		12	1.2
	**Near organs**		5	0.5
	**Lung metastasis**		5	0.5
	**Near organ + lung**		1	0.1
	**Near organ + bone**		1	0.1
	**Lymph node + lung**		1	0.1

*PVT*Portal vein thrombosis* SV*Splenic vein,* SMV*Superior mesenteric vein*, IVC*Inferior vena cava*, HVT*Hepatic vein thrombosis

Nearly half of the patients (54.7%) had a solitary tumor, 158 patients (15.6%) had either intrahepatic or extrahepatic vascular invasion and extrahepatic metastasis was observed in 43 patients (4.3%), mainly in the lymph nodes, lungs, and skeleton. According to BCLC classification, 54 patients (5.3%) were included in BCLC stage 0, 367 (36.2%) in stage A, 375 (37%) in stage B, 178 (17.5%) in stage C and 41 (4%) in stage D. When patients were classified using the HKLC staging system, 299 patients (29.4%) were categorized into HKLC stage I, 145 (14.3%) into stage IIa, 205 (20.2%) into stage IIb, 84 (8.3%) into stage IIIa, 153 (15.1%) into stage IIIb, 60 (5.9%) into stage IVa, 16 (1.6%) into stage IVb, 17 (1.7%) into stage Va and 36 patients (3.5%) into stage Vb. The comparison between BCLC and HKLC staging systems were presented in Table 2 with agreement between both systems regarding early and late stages while in intermediate stage that represent BCLC B, we found that 8.8% of patients were HKLC stage I, 7.7% were stage IIa, and 37.9% were stage IIb, so these patients could be treated with curative therapies rather than TACE and also in advanced BCLC stage C we found that 15.2% of patients were HKLC stage IIb, 0.6% were stage IIIa, and 34.8% were stage IIIb and also they could be treated with curative therapies or TACE rather than systemic therapies.

**Table 2:** BCLC versus HKLC staging of all patients

		HKLC Stage									Total
		I	IIa	IIb	IIIa	IIIb	IVa	IVb	Va	Vb	
**BCLC Stage**	**0**	**42** **77.8%**	**12** **22.2%**	0	0	0	0	0	0	0	54
	**A**	**224** **61%**	**104** **28.4%**	**36** **9.8%**	**3** **0.8%**	0	0	0	0	0	367
	**B**	**33** **8.8%**	**29** **7.7%**	**142** **37.9%**	**80** **21.3%**	**91** **24.3%**	0	0	0	0	375
	**C**	0	0	**27** **15.2%**	**1** **0.6%**	**62** **34.8%**	**60** **33.7%**	**16** **8.9%**	**3** **1.7%**	**9** **5.1%**	178
	**D**	0	0	0	0	0	0	0	**14** **34.1%**	**27** **65.9%**	41
Total		299	145	205	84	153	60	16	17	36	1015

According to the different therapeutic options suggested by both staging systems, in our study, agreement between the HKLC and BCLC staging systems regarding treatments offered was found in 556 (54.8%) of patients while 336 (33.1%) patients received treatment according to BCLC treatment options and 123 patients (12.1%) were already treated according to the HKLC recommendations Table 3. Those 123 patients were included in BCLC stages B (23.6%) and C (76.4%). Certain patients were treated with curative therapies rather than TACE in BCLC stage B and also in advanced BCLC stage C, some of them were treated with curative therapies or TACE rather than systemic therapies Supplementary Table 1.

**Table 3:** Concordance between BCLC and HKLC staging systems regarding treatment modalities received in all patients

Treatment		Within BCLC and HKLC	Within BCLC only	Beyond BCLC (within HKLC only)	Total
**Surgical resection**	**N**	78	0	49	127
	**%**	14%	0%	39.8%	12.5%
**Ethanol injection**	**N**	30	0	0	30
	**%**	5.4%	0%	0%	3%
**Microwave ablation**	**N**	8	0	1	9
	**%**	1.4%	0%	0.8%	0.3%
**Radiofrequency ablation**	**N**	67	0	2	69
	**%**	12.1%	0%	1.6%	6.8%
**Transarterial Chemoembolization**	**N**	203	291	48	542
	**%**	36.5%	86.6%	39%	53.4%
**Sorafenib**	**N**	43	10	0	53
	**%**	7.7	3%	0%	5.2%
**Multiple modalities**	**N**	60	29	23	112
	**%**	10.8%	8.6%	18.7%	11%
**Best supportive care**	**N**	67	6	0	73
	**%**	12.1%	1.8%	0%	7.1%
**Total**		556 (54.8%)	336 (33.1%)	123 (12.1%)	1015

**Fig. 1:**
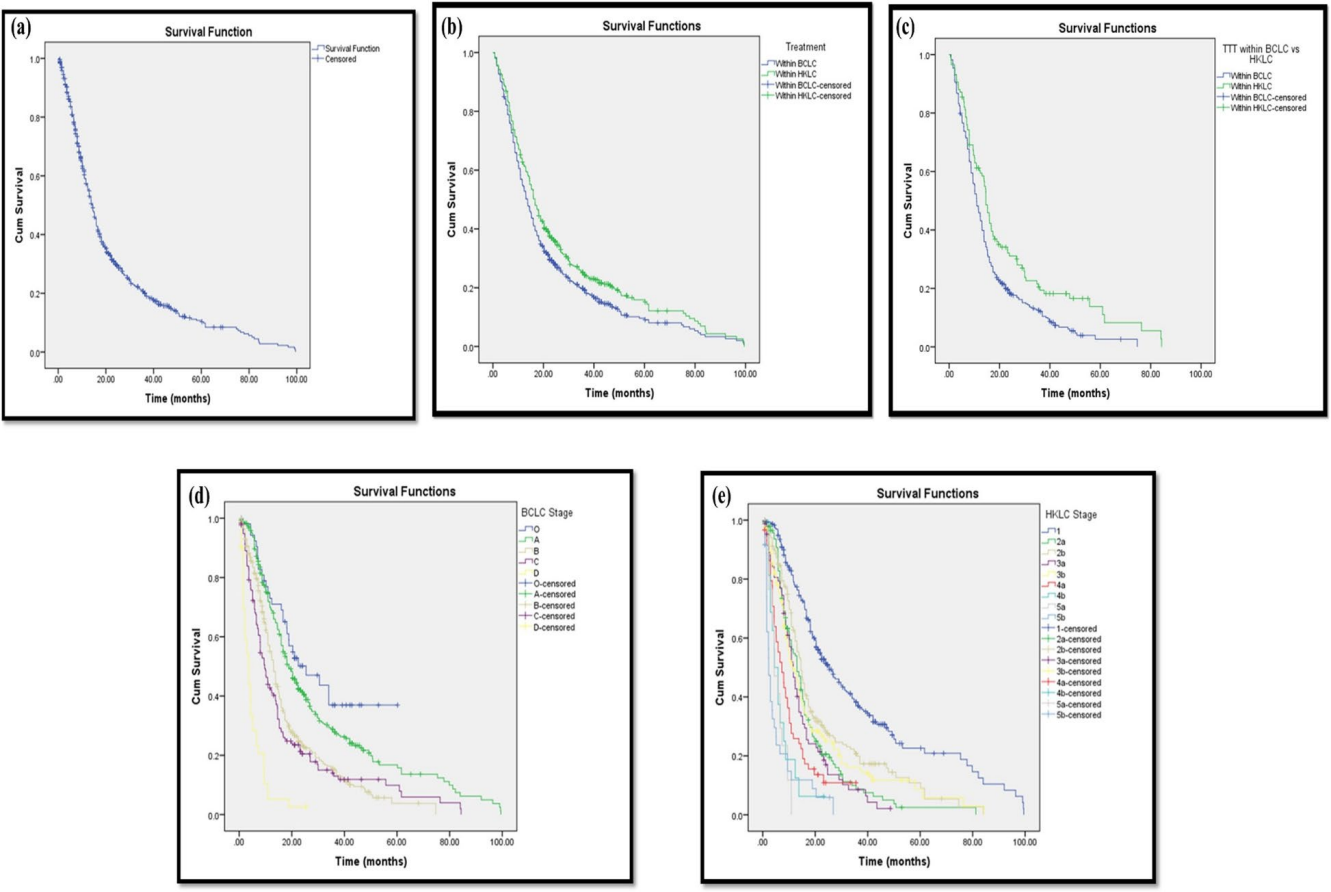
**a** Kaplan-Meier curve for overall survival for all patients. **b** Kaplan-Meier curve for overall survival analysis for all patients regarding treatment according to BCLC versus according to HKLC staging systems. **c** Kaplan-Meier survival curve for BCLC stages B and C patients regarding treatment within BCLC VS. Within HKLC. **d** Kaplan-Meier survival curve for all patients regarding BCLC Stage. **e** Kaplan-Meier survival curve regarding HKLC Stage

At the end of the study, 159 patients (15.7%) were alive while 775 patients (76.4%) were dead. The overall mean survival of all patients was 23.168 months while the median was 14.467 months from the date of diagnosis (Fig. 1a). There was a statistically significant difference in survival regarding different stages of both BCLC and HKLC staging systems (*p*-value 0.0001) (Fig. 1b) (Fig. 1c). Patients’ survival according to HKLC classification (median survival time of 15.2 months) was slightly higher than patients’ survival according to BCLC classification with median survival time of 13.7 months (*p*-value 0.07) (Fig. 1d). Of 459 BCLC stages B and C patients, 123 patients were treated beyond BCLC treatment options according to HKLC recommendations. Their median survival time was 14.6 months which was higher than the 336 patients treated according to BCLC options but not matching HKLC recommendations with median survival time of 12.3 months (*p*-value 0.01) (Fig. 1e).

**Fig. 2:**
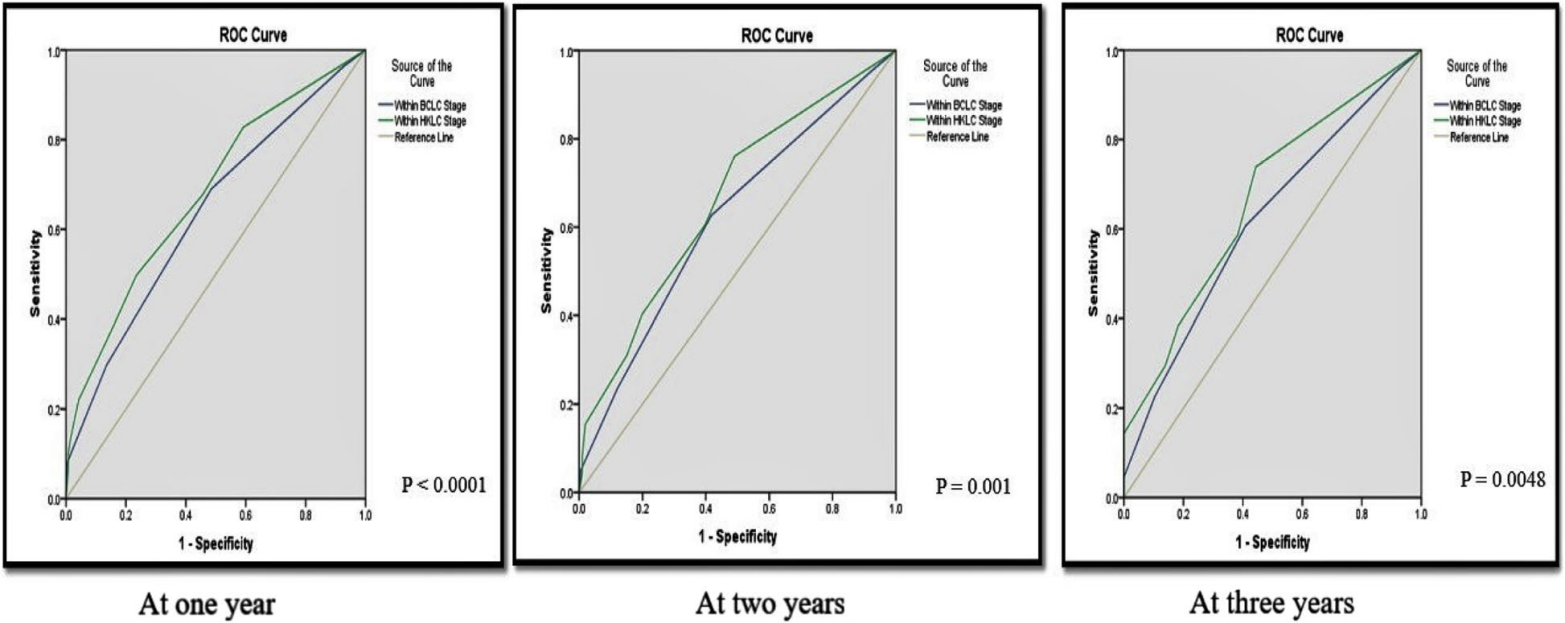
Receiver operating characteristic (ROC) curves for HKLC and BCLC staging systems at one, two & three years

Regarding comparison between the two staging systems, HKLC and BCLC according to ROC curves as shown in Fig. 2, There were statistically significant differences between them at 1, 2 and 3 years. The areas under the receiver operating characteristic curves (AUC) estimated at 1 year were 0.680, 0.635 (*p*-value < 0.0001), at 2 years were 0.661, 0.619 (*p*-value 0.001) and at 3 years were 0.667, 0.619 (*p*-value 0.0048) for the HKLC stage and BCLC Stage, respectively. Higher values (lager AUC) indicate larger separation of classification, indicating better discriminatory ability of the HKLC staging system to predict survival than BCLC staging system and that the HKLC system might be more suitable for predicting prognosis than the BCLC.

In univariate Cox regression analysis, there was increase in the hazard risk of death with the following factors, increase age, male gender, ascites, splenomegaly, hepatitis C, alkaline phosphatase, total and direct bilirubin, AST, creatinine, and AFP levels while there was decrease in hazard risk of death with elevated albumin, prothrombin concentration and INR levels (*P*-value < 0.05). Regarding tumor characteristics, there was increase in the hazard risk of death with all the following factors, multiplicity of focal lesions, increase size, patients who had PVT and patients who had extrahepatic metastasis (*P*-value < 0.05). Also in different patients’ classification, there was increase in the hazard risk of death with upgrading all the following, Child Score, performance status, BCLC stages and HKLC stages (*P*-value < 0.05) Supplementary Table 2. On Multivariate Cox regression analysis, performance status, tumor size, portal vein thrombosis, total bilirubin and INR were the independent prognostic factors affecting OS for studied cases (Table 4).

**Table 4:** Multivariate analysis for detection of the independent factors affecting patients overall survival

	Sig.	Hazard ratio Exp(B)	95.0% CI for Exp(B)	
			Lower	Upper
**Age**	0.097	1.015	0.997	1.034
**Gender**	0.170	0.770	0.529	1.119
**Smoker**	0.620	1.068	0.824	1.383
**Diabetes mellitus**	0.156	1.210	0.929	1.576
**Hypertension**	0.198	0.814	0.595	1.114
**Ascites**	0.580	0.894	0.601	1.329
**Splenomegaly**	0.569	1.089	0.812	1.462
**HBs-Ag**	0.856	0.844	0.135	5.277
**HCV-Ab**	0.559	0.679	0.186	2.484
**Platelets**	0.487	1.001	0.999	1.003
**Total Bilirubin**	**0.004**^**a**^	1.351	1.102	1.656
**Albumin**	0.576	1.069	0.846	1.351
**INR**	**0.025**^**a**^	2.709	1.133	6.476
**Creatinine**	0.370	1.224	0.787	1.903
**AFP**	0.993	1.000	1.000	1.000
**Performance Status**	**0.001**^**a**^	1.556	1.189	2.036
**Lesion Size**	**0.001**^**a**^	1.532	1.202	1.953
**Portal vein thrombosis**	**0.003**^**a**^	1.593	1.170	2.167
**Extrahepatic METS**	0.380	1.268	0.746	2.157

^a^Significant variables in the cox regression

## Discussion

Over the years, numerous staging systems have been developed to address the complex relationship between prognostic factors in HCC patients and to recommend appropriate therapies based on disease stage. However, due to the clinical, biological, and etiological variability among different populations, no single staging system has gained universal acceptance for reliably predicting prognosis or recommending therapeutic approaches [18, 19]. Despite the BCLC staging system being the most widely used and endorsed by organizations such as EASL, EORTC, and AASLD [10, 20, 21], it has its limitations. Even with the 2022 update [22], controversial issues remain, such as the maximum tumor diameter in BCLC-A, and the lack of consideration for transitioning from palliative to curative therapy in TACE responders. Traditionally, the BCLC system recommended TACE for all patients with intermediate-stage tumors (BCLC-B) and did not advance to systemic therapy for TACE non-responders or those with multifocal tumors without metastases until the recent update [18].

In contrast, the HKLC staging system, introduced in 2014, identifies subgroups within intermediate and advanced HCC stages and advocates for more aggressive treatments to improve survival outcomes. This study aimed to compare the prognostic performance of the HKLC and BCLC staging systems in a cohort of 1,015 Egyptian HCC patients and to assess their accuracy in predicting survival. Generally, in most populations, the incidence of HCC increases with age until approximately 75 years, though the median age at diagnosis tends to be younger. In Africa, the median age at diagnosis differs significantly between Egypt (58 years) and other African countries (46 years) [2, 23]. In this study, the majority of patients (52.8%) were smokers or ex-smokers, a known co-factor for hepatocarcinogenesis [24–27].

Globally, around 80% of HCC cases are caused by HBV or HCV, with liver cirrhosis more likely to develop in HCV patients [28]. In many HBV-related HCC cases, particularly in African and Asian populations, cirrhosis is less common, resulting in better-preserved liver function [29, 30]. Therefore, patients, especially Asians, may benefit more from the aggressive treatments proposed by the HKLC system. In our cohort, chronic hepatitis C was the leading cause, reflecting the high prevalence of HCV infection in Egypt and the reduced rate of HBV infection following national infant immunization efforts [31].

Previous studies involving predominantly Western patients with chronic HCV and liver cirrhosis have suggested that the HKLC system may offer superior survival outcomes compared to the BCLC algorithm [18, 32, 33]. However, a multicenter study in France found that the HKLC system did not outperform the BCLC system in prognostic or therapeutic efficacy [34]. Notably, BCLC-B patients classified as HKLC-I/II, for whom the BCLC system recommends only TACE, could benefit from radical therapies as previously reported [35, 36]. Similarly, while surgical resection is contraindicated for BCLC stage C HCC with major vascular invasion according to the BCLC algorithm, it has led to long-term survival in a subset of such patients [37, 38]. BCLC stage C patients with intrahepatic venous invasion, classified as HKLC-II, could also benefit from radical therapies. Recent studies have confirmed that liver resection provided acceptable outcomes among selected patients with BCLC stage B and C HCC [39, 40]. BCLC stage C patients, classified as HKLC-III, could also achieve survival benefits from TACE, as previously observed [41–43].

This study also evaluated the ability of the HKLC and BCLC staging systems to discriminate survival across different stages. Both systems were effective in stratifying patients, consistent with findings from other studies [19, 44–46]. For further analysis, established statistical methods such as the DeLong test and AUC were used to assess the prognostic capabilities of the staging systems. The DeLong test, which measures discrimination between staging systems, yielded significant results [47]. AUC at 1, 2, and 3 years also differentiated patients with varying prognoses for overall survival. Our results, in line with other studies, assigned the HKLC system a higher score compared to the BCLC system [19, 44–46].

Several factors may contribute to the superior prognostic accuracy of the HKLC system. For instance, patients with mild tumor-related symptoms have a better prognosis and may benefit from aggressive therapies, yet the BCLC system categorizes these patients as having at least advanced HCC. Additionally, the HKLC system accounts for differences in prognosis between patients with main portal trunk invasion and those with smaller vascular branch involvement, a distinction not made by the BCLC system [45, 48]. Studies from Taiwan and Italy also suggest that more aggressive treatments than those recommended by the BCLC system could improve outcomes for each BCLC stage [49, 50]. Our results indicate that hepatic resection in a carefully selected subgroup of advanced HCC patients could yield substantial survival benefits. TACE may also provide survival advantages in some BCLC-C/HKLC-III patients in agreement with other studies [16, 19, 45].

Moreover, studies have shown that patients with preserved liver function, even those with multiple tumors, may achieve better survival with hepatic resection compared to nonsurgical treatments [51, 52]. The HKLC system’s ability to identify patients suitable for more aggressive treatments is one of its most significant features. Although the BCLC system has reasonably good discriminatory power, the HKLC system is significantly better at stratifying HCC patients into different prognostic groups [17]. Studies by Liu et al. and others have demonstrated that patients treated according to the HKLC staging system have better overall survival than those treated according to the BCLC scheme [45]. However, Li et al. found the BCLC system to be a better prognostic model than the HKLC system, even among a predominantly HBV-related HCC population in Asia [53, 54]. This discrepancy may be due to the heterogeneity of HCC in terms of clinical characteristics, biological nature, etiology, and pathophysiology across different populations. Additionally, the Child-Turcotte-Pugh (CTP) classification, integral to both BCLC and HKLC systems, may not be sensitive enough, particularly when liver function is well-preserved [55].

These findings suggest that while the BCLC system, which is simpler and more intuitive, should be applied in all HCC cases, the HKLC system can provide valuable information for managing patients, especially in the intermediate stages (BCLC B & C). Identifying independent predictors of survival for HCC patients is also essential. In this study, pretreatment variables were analyzed using univariate and multivariate methods. Several commonly available clinical, laboratory, and tumor parameters were statistically significant in univariate analysis. Multivariate analysis confirmed that ECOG performance status, tumor size, portal vein thrombosis, total bilirubin, and INR are independent predictors of survival. Treatment strategies for HCC are primarily determined by tumor size, number, liver function, and performance status, parameters that have been consistently supported by other studies as significant predictors [56–59].

This study has some limitations, including its single-center, retrospective design, which may introduce bias and limit the ability to draw definitive conclusions. Prospective, multicenter validations are needed to address these issues.

## Conclusion

In conclusion, while both the BCLC and HKLC staging systems are effective in predicting and distinguishing the prognosis of HCC, the HKLC classification demonstrated slightly better prognostic performance compared to the BCLC system. This suggests that the HKLC system may offer a survival advantage by expanding treatment options for patients with intermediate-stage HCC, specifically those classified under BCLC B and C.

## Supplementary Information

Supplementary Material 1.

## Data Availability

Available upon request from the corresponding author.
