# Supplementary material for: Prognostic performance of Hong Kong Liver Cancer with Barcelona Clinic Liver Cancer staging systems in hepatocellular carcinoma
Source: BMC Gastroenterol. 2024 Sep 18;24:318. doi: 10.1186/s12876-024-03387-5 (PMC11409554; doi:10.1186/s12876-024-03387-5)
Supplement: Supplementary file 1 — Supplementary Material 1. [file 12876_2024_3387_MOESM1_ESM.docx]

**Supplementary table 1: *Staging and treatment type in patients treated beyond BCLC but according to HKLC recommendations***

|  | | **HKLC Stage** | | | | | | | | | **Total**  **(123)** |
| --- | --- | --- | --- | --- | --- | --- | --- | --- | --- | --- | --- |
|  |  | **I** | **IIa** | **IIb** | **IIIa** | **IIIb** | **IVa** | **IVb** | **Va** | **Vb** |  |
| **BCLC Stage** | **B** | 6 | 2 | 18 | 0 | 3 | 0 | 0 | 0 | 0 | **29**(23.6%) |
|  | **C** | 0 | 0 | 26 | 1 | 67 | 0 | 0 | 0 | 0 | **94**(76.4%) |
| **Treatment type** | **Resection** | 1 | 0 | 23 | 0 | 25 | 0 | 0 | 0 | 0 | **49**(39.8%) |
|  | **TACE** | 0 | 0 | 9 | 0 | 39 | 0 | 0 | 0 | 0 | **48**(39.1%) |
|  | **Multiple** | 5 | 2 | 9 | 1 | 6 | 0 | 0 | 0 | 0 | **23**(18.7%) |
|  | **RFA** | 0 | 0 | 2 | 0 | 0 | 0 | 0 | 0 | 0 | **2**(1.6%) |
|  | **MWA** | 0 | 0 | 1 | 0 | 0 | 0 | 0 | 0 | 0 | **1**(0.8%) |

*TACE, Transarterial Chemoembolization; RFA, Radiofrequency ablation; MWA, Microwave ablation*

**Supplementary table 2*: Univariate COX regression of prognostic factors of overall survival for all patients:***

|  | **Sig.** | **Hazard ratio Exp (B)** | **95.0% CI for Exp (B)** | |
| --- | --- | --- | --- | --- |
|  |  |  | **Lower** | **Upper** |
| Age | 0.001 | 1.015 | 1.006 | 1.024 |
| Gender | 0.024 | 1.252 | 1.252 | 1.522 |
| Smoking | 0.285 | 1.080 | 0.938 | 1.245 |
| DM | 0.307 | 0.922 | 0.789 | 1.077 |
| HTN | 0.822 | 1.020 | 0.858 | 1.212 |
| Ascites | 0.000 | 1.761 | 1.474 | 2.104 |
| Splenomegaly | 0.038 | 1.192 | 1.010 | 1.406 |
| HBs-Ag | 0.141 | 0.515 | 0.213 | 1.245 |
| HCV-Ab | 0.005 | 2.973 | 1.399 | 6.320 |
| ALT | 0.068 | 1.003 | 1.000 | 1.005 |
| AST | 0.003 | 1.003 | 1.001 | 1.005 |
| Alkaline Phosphatase | 0.025 | 1.002 | 1.000 | 1.004 |
| GGT | 0.174 | 1.002 | 0.999 | 1.006 |
| Hb | 0.512 | 0.986 | 0.944 | 1.029 |
| WBCs | 0.035 | 1.038 | 1.003 | 1.074 |
| Platelets | 0.838 | 1.000 | 0.999 | 1.001 |
| Total Bilirubin | 0.000 | 1.315 | 1.250 | 1.384 |
| Direct Bilirubin | 0.000 | 1.374 | 1.274 | 1.481 |
| Albumin | 0.001 | 0.822 | 0.732 | 0.923 |
| Prothrombin Conc. | 0.019 | 0.993 | 0.987 | 0.999 |
| INR | 0.000 | 2.927 | 2.073 | 4.133 |
| Urea | 0.122 | 1.007 | 0.998 | 1.017 |
| Creatinine | 0.010 | 1.509 | 1.106 | 2.060 |
| AFP | 0.028 | 1.000 | 1.000 | 1.000 |
| Lesion Number Multiple | 0.000 | 1.434 | 1.238 | 1.661 |
| Size ≤ 2 | 0.000 |  |  |  |
| Size (3-5) | 0.030 | 1.506 | 1.040 | 2.179 |
| Size (>5) | 0.000 | 2.459 | 1.706 | 3.543 |
| PV thrombosis | 0.000 | 1.485 | 1.233 | 1.789 |
| Extrahepatic Metastasis | 0.000 | 1.982 | 1.423 | 2.761 |
| Child Score | 0.000 |  |  |  |
| Child Score (A6) | 0.018 | 1.233 | 1.037 | 1.466 |
| Child Score (B7) | 0.000 | 1.916 | 1.569 | 2.341 |
| Child Score (B8) | 0.000 | 1.820 | 1.356 | 2.442 |
| Child Score (B9) | 0.009 | 1.854 | 1.164 | 2.952 |
| Child Score (C10) | 0.000 | 9.302 | 5.949 | 14.547 |
| Child Score (C11) | 0.000 | 9.066 | 4.633 | 17.743 |
| Child Score (C12) | 0.001 | 6.690 | 2.135 | 20.965 |
| Child Score (C13) | 0.000 | 35.670 | 4.886 | 260.424 |
| ECOG PS | 0.000 |  |  |  |
| ECOG PS (1) | 0.000 | 1.913 | 1.620 | 2.260 |
| ECOG PS (2) | 0.000 | 4.192 | 2.728 | 6.441 |
| ECOG PS (3) | 0.000 | 5.520 | 3.029 | 10.058 |
| BCLC Stage | 0.000 |  |  |  |
| BCLC Stage (A) | 0.141 | 1.335 | 0.909 | 1.962 |
| BCLC Stage (B) | 0.000 | 2.180 | 1.488 | 3.192 |
| BCLC Stage (C) | 0.000 | 2.553 | 1.713 | 3.805 |
| BCLC Stage (D) | 0.000 | 9.552 | 5.857 | 15.579 |
| HKLC Stage | 0.000 |  |  |  |
| HKLC Stage (2a) | 0.000 | 2.382 | 1.887 | 3.008 |
| HKLC Stage (2b) | 0.000 | 1.815 | 1.465 | 2.250 |
| HKLC Stage (3a) | 0.000 | 2.729 | 2.073 | 3.593 |
| HKLC Stage (3b) | 0.000 | 2.203 | 1.749 | 2.775 |
| HKLC Stage (4a) | 0.000 | 4.001 | 2.918 | 5.484 |
| HKLC Stage (4b) | 0.000 | 6.693 | 3.931 | 11.396 |
| HKLC Stage (5a) | 0.000 | 9.850 | 5.922 | 16.382 |
| HKLC Stage (5b) | 0.000 | 8.258 | 5.669 | 12.030 |

*DM, Diabetes mellitus; HTN, hypertention HBsAg, Hepatitis B surface antigen. HCV-Ab, Hepatitis C virus antibody. ALT, Alanine Aminotrasferase. AST, Aspartate Aminotransferase. GGT, Gamaglutamyl transferase. Hb, Hemoglobin. WBC, White blood cell. INR, international normalized ratio. AFP, Alpha-fetoprotein. ECOG, Eastern Cooperative Oncology Group; PS, Performance Status; PV, Portal vein. BCLC, Barcelona Clinic Liver Cancer; HKLC, Hong Kong Liver Cancer.*
